# Occurrence and hygienic/allergological relevance of mould from point of view of the environmental medicine* 

**DOI:** 10.5414/ALX01296E

**Published:** 2018-09-01

**Authors:** T. Gabrio, U. Weidner

**Affiliations:** Landesgesundheitsamt Baden-Württemberg im Regierungspräsidium Stuttgart (LGA)

**Keywords:** mold, indoor, allergy and respiratory diseases, sensitization, risk assessment

## Abstract

Allergic skin and respiratory diseases range among the most frequent afflictions in industrialized countries. Due to this fact the importance of indoor mold pollution based on dampness is discussed. In a sentinel health study of the State Health Agency (LGA) children attending of 4th grade of a primary school were tested by an in-vitro allergy screening (UniCap 100/Phadia) for the mold allergens mx1 (Penicillium chrysogenum m1, Cladosporium herbarum m2, Aspergillus fumigatus m3 and Alternaria alternata m6). Primarily about 5% of the children were sensitized against molds which are associated with the ambient air. The investigations showed that most of the children were sensitized against Alternaria alternata and concerning the IgE-concentration (kU/l) Alternaria alternata had the highest concentration among the tested allergens. Commonly children with sensitization against molds were polysensitized. It is unclear if the allergy screening against mold mx1 includes molds with indication for indoor mold pollution such as Acremonium spp., Aspergillus penicillioides, Aspergillus restrictus, Aspergillus versicolor, Chaetomium spp., Phialophora spp., Stachybotrys chartarum, Tritirachium (Engyodontium) album und Trichoderma spp. by means of crossreaction. Therefore, such investigations do not admit any conclusion about health problems as a result of indoor mold pollution. At the present state of knowledge exposure measurements of indoor mold pollutions are not possible, at most a semiquantitative assessment. Although it is generally accepted that dwellings with moisture and mold represent a health risk, knowledge about indoor mold pollution and the related health problems is lacking.

*Based on a lecture on the occasion of the 4^th^ German Allergy Congress, Berlin, September 3 – 6, 2009.

German version published in Allergologie, Vol. 33, No. 3/2010, pp. 101-108

## Introduction 

In highly developed industrial countries allergic skin and respiratory diseases play an important role. For various reasons damages caused by moisture/molds are becoming more important in Germany. This is experienced by the public health services as well as by consumer advice centers, laboratories, dwelling inspectors, craftsmen and so on. Until the beginning of the 1990s the “chemical risk” resulting from formaldehyde, from polycyclic aromatic carbohydrates like it is used e.g. in parquet adhesives, from pentachlorophenol in wood preservatives or from polychlorinated biphenyls in diluents was considered extremely high. Today, the biological risk, especially the one resulting from mold infestation in dwellings, kindergartens, schools or working places, is considered to be particularly dangerous. There are several objective reasons to assume that indoor mold infestation has been increasing in the past years, although data to approve this assumption are lacking. Furthermore, it is not absulutely clear yet which should be the exact parameter for the existence and relevance of a moisture/mold damage. The German Federal Environmental Agency [1, 2] and the Health Agency of the Federal State of Baden-Wuerttemberg [3, 4] have developed criteria for classification of visible mold damages according to their size; however, these criteria are not applicable to invisible damages. These criteria neither allow conclusions for the exposure of persons within the room nor for any health risks these persons may face. 

Among others, the following factors can be reasons for increasing moisture/mold damages:

- the building fabric has become denser in order to save energy,
- people make inadequate efforts to save energy, e.g. by heating only when they are in their dwelling,
- new windows are installed without the complete hull of the building being insulated,
- constructional defects increase, because buildings become more and more complicated and are built faster and faster; furthermore, cost pressure is increasing,
- the building fabric becomes more and more defective, particularly in kindergartens and schools, but also in dwellings, because buildings are maintained inadequately,
- extreme weather, like intense rain, becomes more frequent due to the climate change,
- indoor moisture increases due to altered usage behavior,
- knowledge about adequate usage behavior (ventilation and heating habits, furniture) is not transferred from one generation to the next,
- financial resources for adequate heating and usage behavior are lacking.

Apart from the highly probable objective increase in indoor moisture/mold damages the fact that the subject “indoor mold infestation” is being presented with the tenor to be very dangerous in the media plays an important role in public opinion making. Furthermore, some laboratories, companies and unfortunately also some physicians try to cash in on people’s fear. Frequently, affected persons are being unsettled by the still lacking knowledge about molds and health. It is well known that a risk is considered particularly high when the respective scientific knowledge is low. 

Since 1992 the allergy state of school children in Grade 4 is being assessed using in vitro allergy screenings and a questionnaire by the Health Agency of the German Federal State of Baden-Wuerttemberg (LGA). In the questionnaire parents provide information on age, social environment, existing respiratory diseases or allergies etc. of their children. The evaluation of these questionnaires suggests a relationship between the existence of mold infestation and allergies/respiratory diseases. Therefore, sensitization to molds is being tested since 1997/98 using in vitro allergy screenings. It was also in this period that the mycologic laboratory was established in the LGA. 

## Characteristics and occurrence of molds 

**Figure 1.**
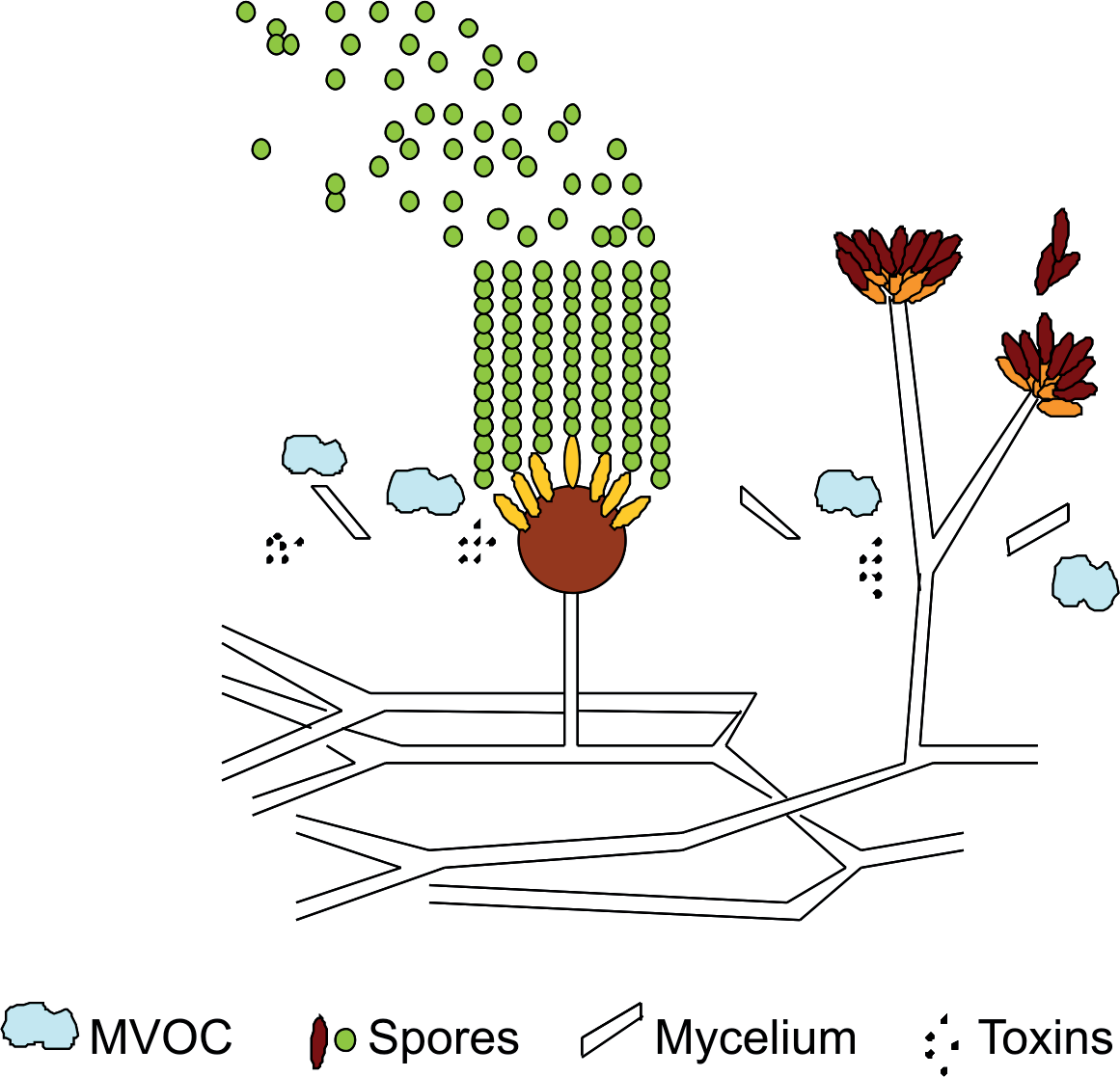
Illustration of 2 mold species.

Molds are eukaryotes. In the biosphere they undertake the important task of decomposing organic material. There are probably more than 100,000 species of molds. Of all biological material on earth approximately 5% are molds. The most important prerequisite for molds is moisture. With regard to nutrient requirements molds are extremely undemanding. Depending on the species, molds can grow at temperatures below 0 °C and above 37 °C. In cases of mold infestation it must be assumed that mold spores, mycelial fragments, toxins and microbial volatile organic compounds (MVOC) are present in the ambient air (Figure 1). Furthermore, it must be presumed that in cases of moisture or mold damages also bacteria, in particular actinomycetes, as well as metabolites and cell fragments, like endotoxins, β-glucans and fragments of mold spores or bacteria are present. The occurrence of molds is frequently accompanied by increased numbers of mites and ameba. If a moisture or mold damage is present, it also must be assumed that the affected persons are exposed to a variety of biologic substances. Frequently it is unknown that certain impacts on health, like for example toxic or allergenic effects, can emanate from both viable and nonviable mold spores. Below are the reasons why current knowledge is not sufficient to evaluate mold exposure [7]:

- It remains unclear which of the different biological substances has effects on people’s health.
- It is still uncertain which medium (air, dust, construction material etc.) leads to a relevant exposure.
- For practical reasons usually short-term measurements are carried out; however, these are not representative.

Molds are always being present in our natural environment. In Central Europe approximately 200 mold species are estimated to be present indoors and outdoors. Most of the different mold species are associated with certain sources, for example

- Cladosporium herbarum, Alternaria alternata, Botrytis cinerea – vegetation,
- Aspergillus fumigatus – composting, rotting of plant material,
- many Penicillium species – perishing foods, decomposing foods, waste, bio-waste,
- Stachybotrys chartarum, Acremonium spp. – very moist, cellulosic construction material,
- Phialophora spp., Engyodontium album – moist plaster,
- Aspergillus penicillioides, Aspergillus restrictus, Eurotium spp., Wallemia sebi – cellulosic material with slightly increased moisture,
- Aspergillus versicolor, Chaetomium spp., Trichoderma spp. – moist building fabric,
- Eurotium spp. – moist leather (shoes etc.), animal husbandry,
- Wallemia sebi, Eurotium spp. – animal caging with litter.

Depending on vegetation, molds are always present in the ambient air. In Central Europe the concentration of molds in the ambient air is approximately 100 cultivable mold spores per m3 in winter and several thousand in summer. 

In cases of indoor moisture damage the following “indicating” mold species are frequently present: Acremonium spp., Aspergillus penicillioides, Aspergillus restrictus, Aspergillus versicolor, Aureobasidium pullulans, Chaetomium spp., Phialophora spp., Stachybotrys chartarum, Tritirachium (Engyodontium) album and Trichoderma spp. 

## Detection of molds 

If a mold infestation is visible, it is usually not necessary to determine the species of the present mold. If it is uncertain whether a mold infestation or only a discoloration of the material in question is present, analysis using the adhesive tape technique is recommended: about 2 cm of an approximately 5 cm-long transparent adhesive tape are slightly pressed on the spot in question and then torn away. Under the microscope the present mold species can be identified by their morphological structures. If an invisible infestation is suspected or if a physician thinks an exposure assessment is necessary due to a patient’s medical condition, air sampling for mold spores, analysis of particles and/or material samples [7, 8] can be reasonable. For the proper interpretation of these examinations usually the species of the present molds have to be determined, as their effects on health as well as their association with certain sources are normally specific. To assure the analytical quality of laboratories for environmental mycology the interlaboratory test “Differentiation of indoor- and food-relevant molds” was established by the LGA [9, 10]. The quantitative determination and the qualitative detection of the corresponding microbiological parameters are afflicted with high error rates and require a high qualification of the investigating laboratories. The still applied sedimentation method with a collection plate is inappropriate in this concern [11]. 

## Hygienic effects of molds 

The following symptoms are suspected to be resulting from molds:

- sensitization and allergies,
- irritative effects – mucous membrane irritation (MMI),
- chronic bronchitis,
- infections,
- allergic bronchopulmonary aspergillosis,
- intoxications,
- unpleasant odors,
- unspecific feeling of illness [7].

**Figure 2.**
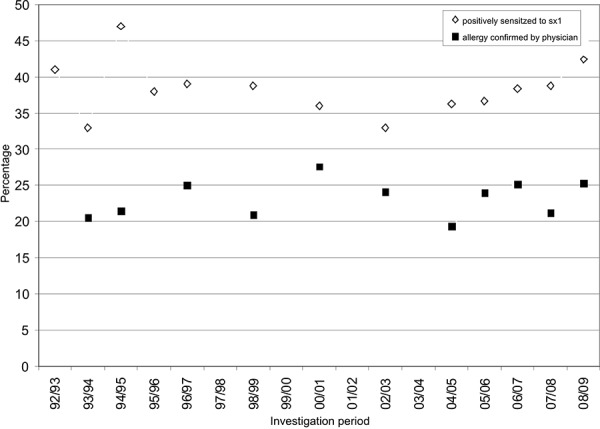
Percentage of positive sx1 test and confirmed allergy in the investigational collective of Ravensburg in the investigation period 1992/93 – 2008/09.

Of these effects on health the allergenic effect of molds is the most important one. Since 1992 an in vitro allergy screening is carried out in 4th grade children with a median age of 10 years and a relatively uniform distribution of boys and girls in the framework of a sentinel health study of the State Health Agency of Baden-Wuerttemberg (LGA). An exposure and effect monitoring was carried out annually in the beginning and every 2 years later. In winter in vitro allergy screenings using the sx1 test were carried out to test whether a sensitization to frequent airborne allergens existed. The atopy screening is composed of Dermatophagoides pteronyssinus (d1), cat dander (e1), dog dander (e5), timothy grass (g6), rye (g12), birch (t3), mugwort (w6) and Cladosporium herbarum (m2) (Phadiatopttest, Phadia AB, Uppsala, Sweden). In the past 17 years no significant increase in sensitization could be detected by this test. In the investigated region of Ravensburg sensitization fluctuated between 33% and 47%. There was a very slight increase in the proportion of parents who indicated in the questionnaire that a physician had diagnosed an allergy in their child (Figure 2). Since 1997/98 also a testing for mold allergens is being carried out in the framework of this project. This testing is carried out using the mold allergen mix mx1 by the manufacturer Phadia AB (Penicillium chrysogenum m1, Cladosporium herbarum m2, Aspergillus fumigatus m3 and Alternaria alternata m6) or mx2 (Penicillium chrysogenum m1, Cladosporium herbarum m2, Aspergillus fumigatus m3, Candida albicans m5, Alternaria alternata m6 and Helminthosporium halodes m8). 

**Figure 3.**
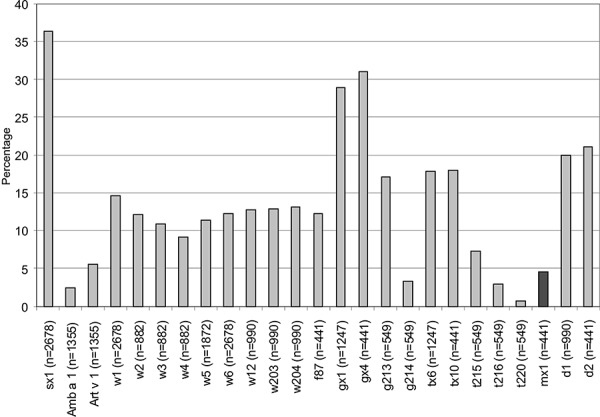
Comparison of serum prevalence to the tested allergens (in parantheses: number of tested samples) in the framework of the sentinel health study in the investigation period 2004 – 2009: sx1 mixed allergen, Amb a 1 – Ambrosia major allergen, Art v 1 – mugwort major allergen, w1 – Ambrosia artemisiifolia, w2 – Ambrosia psilostachya, w3 – Ambrosia trifida, w4 – Ambrosia acanthicarpa (flatspine burr ragweed), w5 – Artemisia absinthium (wormwood), w6 – Artemisia vulgaris (common mugwort), w12 – golden rod, w203 – rape, w204 – sunflower, f87 – melon, gx1 – grasses/early flowering plants, gx4 – grasses/late flowering plants, tx6 – trees, tx10 – trees, g213 – rPhl p 1, rPhl p 5b (mixture of recombinant timothy grass major allergens), t215 – rBet v 1 (recombinant birch major allergen), t216 – rBet v 2 (recombinant birch profilin), t220 – rBet v 4 (recombinant birch minor allergen, Ca binding), g214 – rPhl p7, rPhl p12 (mixture of recombinant pan allergens of timothy grass, CBP, profilin), mx1 – mold (mixture), d1 – Dermatophagoides pteronyssinus (house dust mite), d2 - Dermatophagoides farinae (house dust mite).

**Figure 4.**
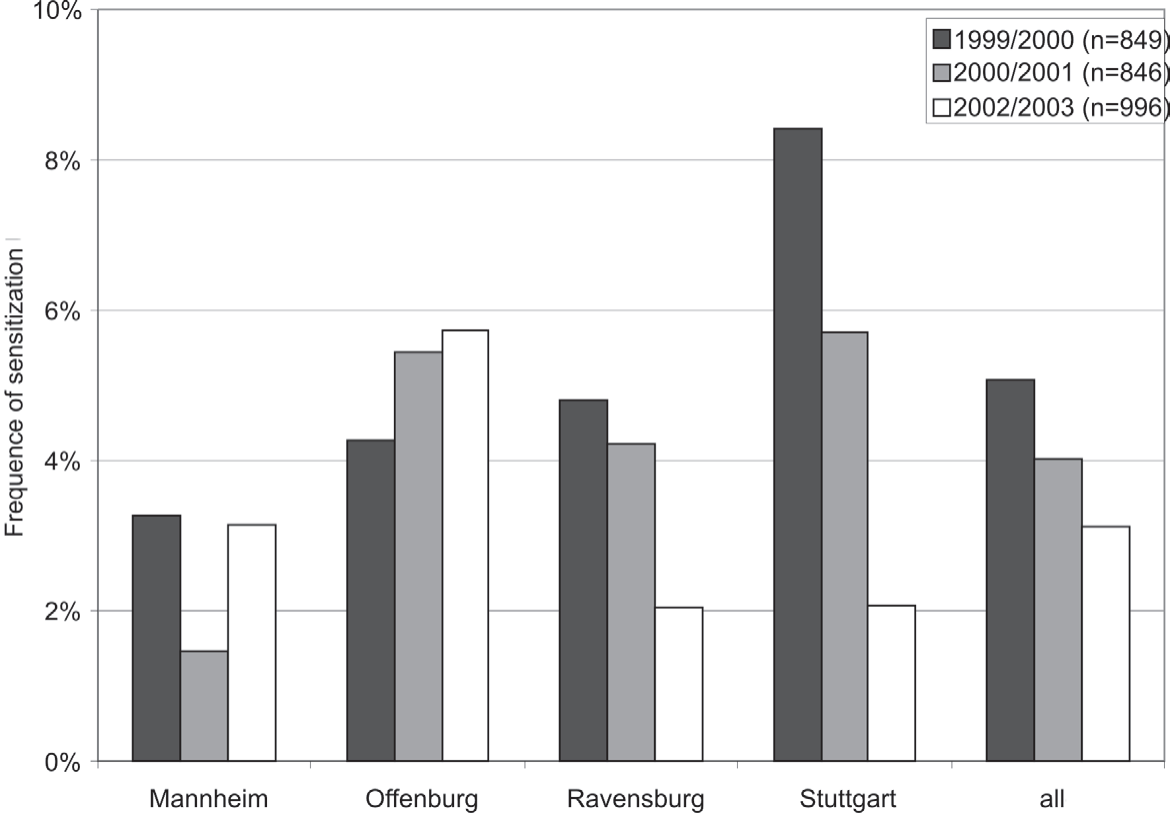
Frequency of sensitization to the mold mixture mx2 in 4 investigational regions in the sentinel health study of the State Health Agency (LGA) of Baden-Wuerttemberg.

In the investigational project “Ambrosia 2004 – 2009” [12] the occurrence of sensitization to these mixed allergens was 5% and thus relatively low as compared to many other airway allergens (Figure 3). In other investigated regions (Figure 4) and in the literature [7] a similar occurrence of sensitization to “molds” was shown. The frequency of sensitization was not consistent between the different investigated regions. This is due to the – in view of the frequency of sensitization – low number of test persons. When interpreting the results it must also be considered that the tested molds Penicillium chrysogenum m1, Cladosporium herbarum m2, Aspergillus fumigatus m3 and Alternaria alternata m6 are rather associated with the ambient air or general hygienic questions and have little or nothing to do with indoor areas. Thus, conventional allergy diagnosis does not allow any conclusions concerning the question of “indoor mold infestation” and occurring allergic symptoms. So far it remains uncertain if and if so which indoor mold species are covered due to cross-reactions when testing for the conventional allergen mixtures. 

**Figure 5.**
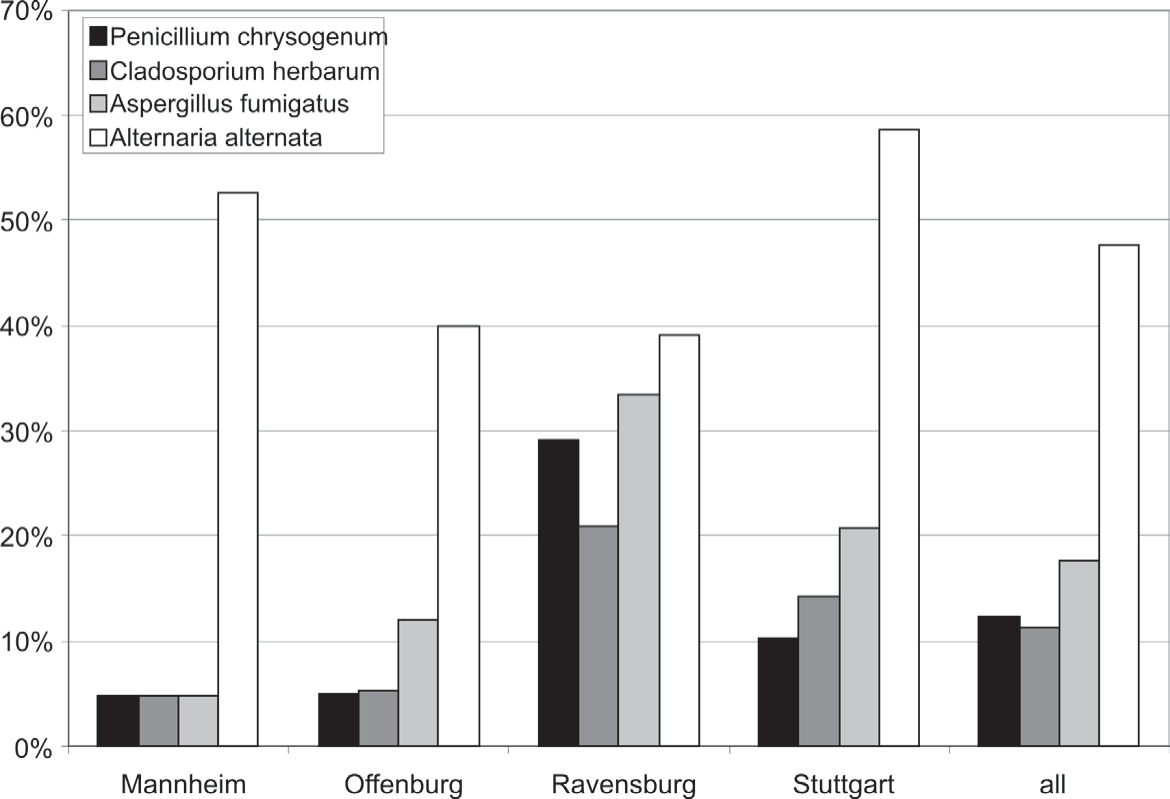
Percentage of tested persons sensitized to the mold allergen mixture mx2 who reacted positive to single allergens (1999/2000).

**Figure 6.**
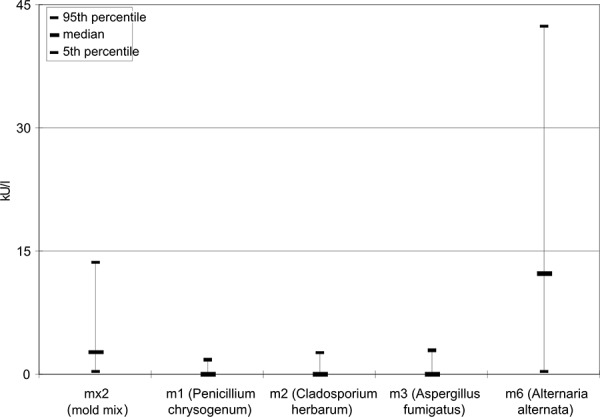
Specific IgE concentrations (kU/l) of children sensitized against the mold allergen mix mx2 (1999/2000).

**Figure 7.**
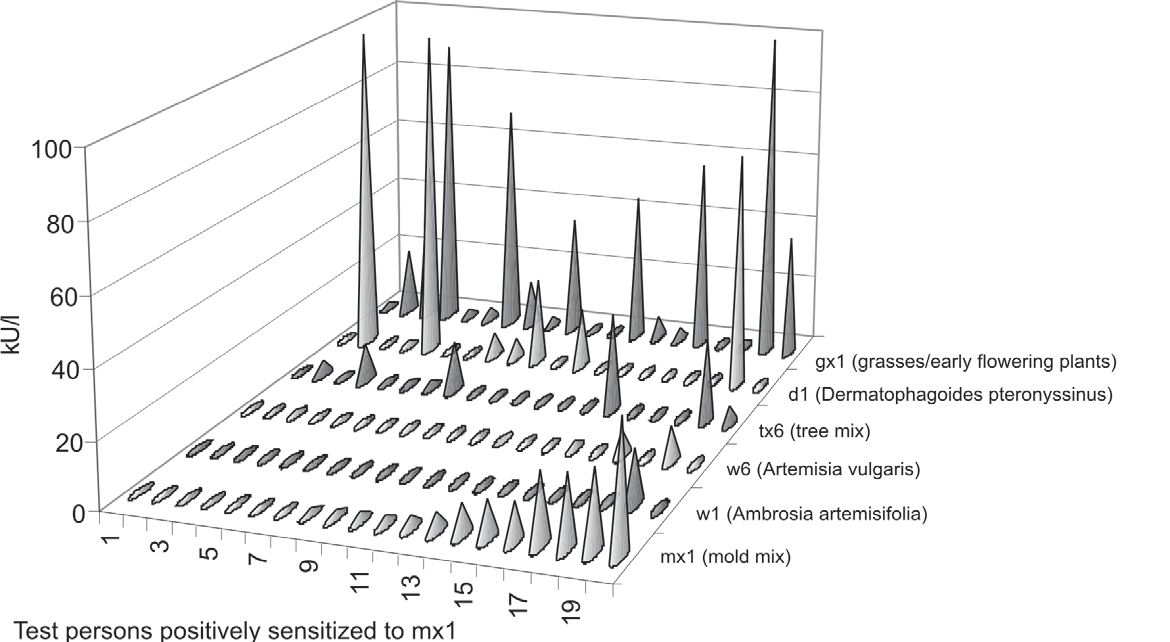
Level of sensitization (kU/l) in the 20 children with positive sensitization to the mx2 test (2006/2007).

In the investigation period 1999/2000 those children who reacted positive in the in vitro allergy screening mx2 test were tested for a sensitization to the single allergens Penicillium chrysogenum m1, Cladosporium herbarum m2, Aspergillus fumigatus m3 and Alternaria alternata m6 (Figure 5). This investigation showed that children were most frequently sensitized to Alternaria alternata m6. Figure 6 shows that also the specific IgE concentration (kU/l) was highest for Alternaria alternata m6. It is also noteworthy that the specific IgE concentration for Alternaria alternata m6 was higher than in the mx2 test. Figure 7 shows that in most persons who are sensitized to molds a polysensitization is present. Furthermore, Figure 7 shows that the children examined in 2006/2007 were not only sensitized to the mold mixture mx1 but normally also to weed, grass or tree pollen or to mites. 

## Discussion and perspectives 

At the present state of knowledge it is difficult to assess the allergologic risk caused by molds exactly, because

- the disposition of affected persons concerning the development of allergy plays an important role,
- the possibilities to diagnose mold allergy, especially in cases of indoor molds, are so far insufficient,
- it remains unclear which indoor mold species are covered due to cross-reactions when testing for the mx1 and mx2 allergens,
- exposure to molds can only be assessed and it remains unclear which “mold components” in which medium are significant in an environmental medical context:
  - the concentration of cultivable and non-cultivable mold spores and bacteria in air, in dust, in or on construction material,
  - the area of material infested with mold spores and bacteria,
  - the concentration of metabolites, cell components and cell fragments of mold spores and bacteria in the air and in dust,
  - the relative moisture of the air or of surfaces of building elements.

**Figure 8.**
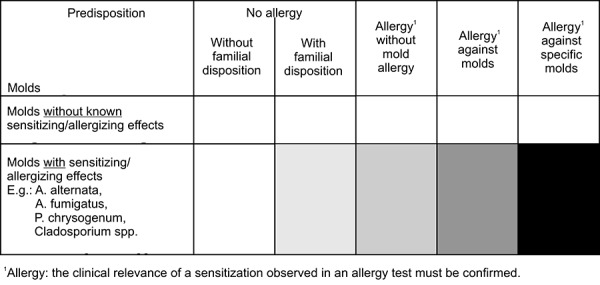
Risk matrix: risk of sensitization/allergization caused by molds (the darker the field the higher the potential health risk) [7, 13, 14].

Wiesmüller et al. [7, 13, 14] developed a risk matrix (Figure 8) that allows to assess the risk of sensitization/allergization; however, it has to be considered that at present the diagnostic possibilities concerning the detection of mold allergies are insufficient. 

Despite the high number of open questions it is generally assumed that moist, moldy rooms represent a health risk. The allergenic potential of molds probably plays the most important role for the general population.
